# Nosocomial Infections among Pediatric Patients with Neoplastic Diseases

**DOI:** 10.1155/2009/721320

**Published:** 2009-10-26

**Authors:** Peninnah Oberdorfer, Natthida Pongwilairat, Charles H. Washington

**Affiliations:** ^1^Department of Pediatrics, Faculty of Medicine, Chiang Mai University, Chiang Mai 50200, Thailand; ^2^Department of Pediatrics, Buddha Chinaraj Hospital, Phitsanulok 6500, Thailand; ^3^Department of Epidemiology, Bloomberg School of Public Health, Johns Hopkins University, Baltimore, MD 21250, USA

## Abstract

*Background*. Pediatric patients with neoplastic diseases are more likely to develop nosocomial infections (NIs). NIs may prolong their hospital stay, and increase morbidity and mortality. *Objectives*. The objectives of this study were to determine: (1) the incidence of NIs, (2) sites of NIs, (3) causal organisms, and (4) outcomes of NIs among pediatric patients with neoplastic diseases. *Methods*. This study was a prospective cohort study of pediatric patients with neoplastic diseases who were admitted to the Chiang Mai University Hospital, Thailand. *Results*. A total of 707 pediatric patients with neoplastic diseases were admitted. Forty-six episodes of NIs in 30 patients were reported (6.5 NIs/100 admission episodes and 7 NIs/1000 days of hospitalization). Patients with acute lymphoblastic leukemia had the highest number of NIs (41.3%). The most common causal organisms were gram-negative bacteria (47.1%). Patients who had undergone invasive procedures were more likely to develop NIs than those who had not (*P* < .05). The mortality rate of patients with NIs was 19.6%. *Conclusion*. Pediatric patients with neoplastic diseases are more likely to develop NIs after having undergone invasive procedures. Pediatricians should be aware of this and strictly follow infection control guidelines in order to reduce morbidity and mortality rates related to NIs.

## 1. Introduction

The recent medical developments, including the increased use of chemotherapy drugs, white blood cell stimulants, and broad spectrum antibiotics, have improved the prognosis and life span of pediatric patients with neoplastic diseases. Consequently, these patients often face lengthy periods of low immunity, undergo longer hospital stays, and there is a greater chance that they will require central venous catheterizations, urinary catheterizations, endotracheal intubations, and intravenous feeding tubes. These factors moreover put patients at an increased risk of contracting nosocomial infections (NIs) and substantially increase morbidity and mortality rates as well as treatment costs [1–3].

Nosocomial infections in patients with malignancies can be caused by bacteria, fungi, and viruses and can occur in the bloodstream; urinary, respiratory, and digestive tracts; as well as soft tissues [2]. The previous studies have been done among both adult and pediatric patients with neoplastic diseases reporting a high risk of NIs [4–7] and showing incidence rates of NIs ranging from 1.08 to 1.77 times/100 days of hospitalization [8–11].

In addition, the previous studies among pediatric patients with neoplastic diseases found that NIs were associated with the use of devices [6–11]. For example, a US surveillance among pediatric patients with neoplastic diseases found that bacteremia occurred 6.6 times/1000 days of central venous catheterization, urinary tract infections 4 times/1000 days of urinary catheterization, and pneumonia 2.9 times/1000 days of endotracheal intubation [7]. Causal organisms related to NIs vary according to settings and study populations. In Germany, Simon et al. identified gram-positive bacteria as the common causal organism of NIs (83.3%) among pediatric patients with central venous catheterization [10]. In contrast, a study by Frank et al. in Israel found gram-negative bacteria (54.3%) more common than gram-positive bacteria (36.6%) among children and adolescents in intensive care settings [12]. 

Most NIs have a significant effect since they lengthen hospital stays, increase mortality, and increase complications [8–11]. At present, studies of NIs in pediatric patients with neoplastic diseases are under reported in Thailand.

## 2. Objectives

To determine (1) the incidence of NIs among pediatric patients with neoplastic diseases, (2) sites of NIs, (3) causal organisms, and (4) outcomes of NIs.

## 3. Methods

### 3.1. Patients and Setting

The study was conducted in the 32-bed pediatric hematology/oncology ward of the Chiang Mai University Hospital, Chiang Mai, Thailand. Patients in this ward are up to 15 years old and all have neoplastic diseases. The patients received chemotherapy regimens based on recommendations by the Thai Pediatric Oncology Group. Antibiotic and antifungal prophylaxes are not routinely provided. We excluded those patients who (1) had fever of unknown origin, since we could not find any other clinical or radiological signs of infection as well as isolate any causative organisms and therefore could not classify them as having an NI with certainty, (2) received any antibiotic prophylaxis, and (3) had viral-related illness diagnosed by clinicians. 

### 3.2. Surveillance Procedures of NIs and Case Definitions

We conducted a prospective cohort study during December 2005 and May 2006. The clinical symptoms of each patient were monitored daily from admission until hospital discharge by pediatricians and nurses. Data were obtained from medical records and nurse notes. The findings were recorded during admission on a data extraction form that included demographic data, discharge diagnoses, intrinsic risk factors, extrinsic risk factors, causal organisms, and treatment outcomes. The definitions for NIs were based on the criteria outlined by the US Centers for Disease Control and Prevention in 2004 [13]. Neoplastic diseases in pediatric patients were classified as follows: hematologic neoplasia (acute lymphoblastic leukemia (ALL), acute myeloid leukemia (AML), non-Hodgkin's lymphoma, Hodgkin's disease), solid tumors (bone tumors, rhadomyosarcoma, central nervous system tumors, neuroblastoma, and Wilm's tumor), and others (Schwanoma, hepatoblastoma, and lymphangioma). 

### 3.3. Analysis of Data

The data were analyzed by calculating NI rates per 1000 days of hospitalization and per 100 admittances. The relationship between NI rates and various extrinsic factors was analyzed using a chi-square test when there was a need to compare proportional data, using a level of 95% confidence interval. 

## 4. Results

### 4.1. Population Characteristics

We collected data of 707 admissions (6561 days of hospitalization) during the study period (Table 1). Fifty-four percent were males with a mean age of 6.9 years (range: 3 months–15 years; SD = 4 years). The most common neoplastic disease on admission was ALL (59%). Eighty-seven percent of patients had leukopenia, 52.2% had an absolute neutrophil count of less than 500/mm^3^, and 58.7% had thrombocytopenia.

### 4.2. Rates of NIs

Nosocomial infections were reported among 46 admissions (6.5/100 admission episodes; 7 episodes/1000 days of hospitalization). There were 13 episodes of urinary tract infections per 1000 days of urinary catheterization, 21 episodes of pneumonia per 1000 days of endotrachial intubation, and no episodes of bacteremia among patients who had central venous catheterization. Episodes of NIs were most frequent among patients with ALL (41.3%), and patients with AML (34.8%).

### 4.3. Sites of NIs

The most common sites of NIs were the blood stream (30.5%) and the ear/nose/throat (19.6%) (Table 2).

### 4.4. Causal Organisms of NIs

Causal organisms of NIs were identified in 34 episodes (73.9%). The most common were gram-negative bacteria (47.1%), followed by gram-positive bacteria (29.4%), and fungi (14.7%) (Table 3).

### 4.5. Procedures Related to NIs

Patients who developed NIs were more likely to have had endotracheal intubation (mean duration: 10.2 days; range: 1–15 days), urinary catheterization (mean duration: 5.8 days; range: 1–10 days), nasogastric tube (mean duration: 4.9 days; range: 1–17 days), and central venous catheterization (mean duration: 1.7 days; range: 1-2 days) (*P* < .001) (Table 4). 

### 4.6. Outcomes of NIs

The mean time from admittance until time of diagnosis of an NI was 22 days (range: 2–126 days; SD: 23 days). The majority of NIs (74%) occurred between the 2nd and the 30th day of hospitalization.

Nine patients died (19.6%): 4 with ALL (44.4%), 4 with AML (44.4%), and 1 with an astrocytoma brain tumor. Four patients (44.4%) had bacteremia, 3 (33.4%) had soft tissue infections, 1 (11.1%) had pneumonia, and 1 had both bacteremia and pneumonia. Three patients (33.4%) were infected with gram-positive bacteria, 2 (22.2%) with gram-negative bacteria, 2 (22.2%) with fungal organisms, and 1 patient (11.1%) was found to be infected with gram-positive bacteria and a fungal organism. 

## 5. Discussion

A total of 707 admission episodes were included in the study. Forty-six episodes of NIs were reported (the incidence of NIs was 6.5/100 admission episodes; 7 episodes/1000 days of hospitalization). A previous study of pediatric patients with neoplastic diseases in Germany by Simon et al. [10] found a rate of NIs of 5.2 cases per 100 admittances and of 10.8 cases per 1000 days of hospitalization, which is similar to the results obtained by our study. A study of Urrea et al. [11] among pediatric patients with neoplastic diseases in Spain found an NI rate of 1.77 cases per 100 days of hospitalization. The incidence of NIs in our study was relatively low in comparison to the study of Urrea et al. This may be because our study excluded patients with fever of unknown origin and those who had viral infections. In addition, in our study patients appeared to have lower rates of central venous catheterizations than patients in previous studies [5, 10, 11]. Patients with ALL who represented 59% of the sample population had the highest NI rate (41.3%) in our study. Since the percentage of ALL patients in our study was higher than that reported in other studies [10, 11], it could represent a skewed population.

In regards to sites of NIs, most infections in our study occurred in the blood stream (30.5%), as in other studies by Simon et al. [10] (52.5%) and Urrea et al. [11] (55.5%). Regarding causal organisms of NIs, studies from Eastern countries found that gram-negative bacteria were most common, like in our study. Our study found 47.1% gram-negative bacteria and 29.4% gram-positive bacteria while the study by Frank et al. [12] in Israel from 1992 to 2001, which focused particularly on bacteremia in pediatric wards, found 54.3% gram-negative bacteria and 36.6% gram-positive bacteria. However, our study focused on children with neoplastic diseases while Frank's et al. study included the general pediatric population, including intensive care units. Therefore this comparison might not be completely valid. In contrast, studies from European countries were more likely to report gram-positive bacteria to be more common. For example, the study by Simon et al. [10] found up to 83.3% gram-positive and 11.1% gram-negative bacteria, and the study by Urrea et al. [11] reported up to 78.6% gram-positive bacteria. The higher rate of gram-positive bacteria as causal organisms for NIs in European countries could be due to the fact that in European countries central venous catheterization is more common than in Eastern countries, such as in Thailand. In addition, in our study we found 3 cases of *Salmonella enteritidis* infections. *Salmonella enteritidis* is unlikely to be a causal organism of NIs since it is normally found in community settings. We think that these patients may have been colonized with this organism outside the hospital and then developed symptoms when their immunity level was low after chemotherapy. 

Furthermore, our study found that fungal infections accounted for 14.7% of infections, while in the previous two studies by Simon et al. [10] and Urrea et al. [11] no fungal infections were found among pediatric patients with neoplastic diseases. However, in the Israeli study, fungal infections also occurred, most of which were bloodstream infections. Therefore, among pediatric patients with neoplastic diseases who suffer from a low white blood cell count for a long period of time, care should be taken to guard against fungal infections as well.

Concerning procedures that can make patients vulnerable to NIs, we found that endotracheal intubation, nasogastric tube insertion, urinary catheterization and central venous catheterization significantly increased the incidence of NIs (*P*-value < .001). Patients who were exposed to more invasive procedures such as endotracheal intubation (mean duration: 4.9 days) and central venous catheterization (mean duration: 1.7 days) also more quickly developed NIs than patients who were exposed to less invasive procedures such as nasogastric tube insertion (mean duration: 10.2 days). We therefore recommend that the more invasive procedures should be carried out only when necessary in order to reduce the incidence of NIs. 

The strengths of our study are twofold. First, it is a prospective study in which NI episodes were carefully monitored and the data collection carried out according to a given research plan. Second, according to our best knowledge, it is the first study of NI episodes among pediatric patients with neoplastic diseases in Thailand. 

However, our study also has the following limitations. First, the period of data collection was relatively short. A longer period of data collection would be able to provide a clearer picture of NI episodes among this group. Second, care should be taken when comparing the incidence rates of NIs of our study with those of other institutions and countries, since we have excluded patients who had fever of unknown origin and viral related illnesses. These entities are computed in most studies as NIs per CDC criteria. Third, we also have not recorded the types of chemotherapy regimens these patients received as well as their cancer stages, which may have some impact on episodes of NIs. Further studies could address these shortcomings. Fourth, our study also did not investigate the relationship between the incidence of NIs and other intrinsic factors, including the presence of other underlying diseases as well as level of anemia and white blood cell counts.

## Figures and Tables

**Table 1:** Demographic characteristics of study samples.

Demographic characteristics	Number of admissions (%) (*N* = 707)	Number NIs (%) (*N* = 46)
*Gender and age*		
Male : Female	380 (53.7) : 327 (46.3)	27 (58.7) : 19 (41.3)
Mean age (years) ± SD	6.88 ±4	6.97 ±4.92
*Underlying diseases*		
Hematologic neoplasia		
Acute lymphoblastic leukemia	417 (59.0)	19 (41.3)
Acute myeloblastic leukemia	59 (8.4)	16 (34.8)
Non-Hodgkin lymphoma	27 (3.8)	1 (2.2)
Hodgkin disease	11 (1.6)	1 (2.2)
Chronic myeloid leukemia	11 (1.6)	—
Solid tumor		
Bone tumor		
Osteosarcoma	31 (4.4)	1 (2.2)
Ewing's sarcoma	25 (3.5)	—
Neuroblastoma	29 (4.1)	—
Hepatoblastoma	14 (2.0)	—
Retinoblastoma	14 (2.0)	—
Rhabdomyosarcoma	13 (1.8)	—
Wilm tumor	10 (1.4)	1 (2.2)
Hepatoma	7 (1.0)	—
Germ cell tumor	5 (0.7)	1 (2.2)
CNS tumor +	34 (4.8)	6 (13.1)

+: Astrocytoma, Medulloblastoma, Medulloepithelioma, Ependymoma.

**Table 2:** Types of nosocomial infections.

Types of NIs	Total Number of NIs (%) (*N* = 46)	Associated procedures (*N* = 32)
Blood stream	14 (30.5)	ET (5), U (5), C (2)
Ear/nose/throat	9 (19.6)	NG (9)
Soft tissue	6 (13.1)	—
Gastrointestinal tract	6 (13.1)	NG (2)
Urinary tract	5 (10.9)	U (5)
Pneumonia	4 (8.7)	ET (4)
Surgical site	1 (2.2)	—
Meningitis	1 (2.2)	—

ET: endotracheal intubation; U: urinary catheterization; NG: nasogastric tube; C: central venous catheterization.

**Table 3:** Causal organisms of nosocomial infections.

Causal organisms	Number (%) (*N* = 34)
*Gram-negative organisms*	16 (47.1)
* Escherichia coli*	3 (8.8)
* Klebsiella pneumonia*	3 (8.8)
* Salmonella enteritidis*	3 (8.8)
* Pseudomonas aeruginosa*	2 (5.9)
Others+	5 (2.9)
*Gram-positive organisms*	10 (29.4)
*Staphylococcus aureus *	5 (14.7)
Coagulase negative *Staphylococcus *	4 (11.8)
*Enterococcus fecium *	1 (2.9)
*Fungi*	5 (14.7)
* Candida albicans*	3 (8.8)
* Candida tropicalis*	2 (5.8)
*Co-organisms*	3 (8.8)
*Enterococcus fecium * + Coagulase negative *Staphylococcus *	2 (5.9)
*Enterococcus fecium * + *Escherichia coli *	1 (2.9)

+: *Acinetobactor baumanii*, Aeromonas, *Burkholderia cepacia, Enterobactor cloacae, Hemophilus influenza.*

**Table 4:** Comparison of procedures related to nosocomial infections.

Procedures	NIs (%) (total = 46)	Non-NIs (%) (total = 661)	*P*-value
Endotracheal intubation	9 (19.6)	3 (0.4)	*P* < .001
Urinary catheterization	10 (21.7)	6 (0.9)	*P* < .001
Nasogastric tube	11 (23.9)	6 (0.9)	*P* < .001
Central venous catheterization	2 (4.3)	0 (0)	*P* < .001
